# Underlying mechanism of subcortical brain protection during hypoxia and reoxygenation in a sheep model - Influence of α1-adrenergic signalling

**DOI:** 10.1371/journal.pone.0196363

**Published:** 2018-05-29

**Authors:** René Schiffner, Sabine Juliane Bischoff, Thomas Lehmann, Florian Rakers, Sven Rupprecht, Georg Matziolis, Harald Schubert, Matthias Schwab, Otmar Huber, Cornelius Lemke, Martin Schmidt

**Affiliations:** 1 Orthopedic Department, Jena University Hospital—Friedrich Schiller University, Eisenberg, Germany; 2 Department of Neurology, Jena University Hospital—Friedrich Schiller University, Jena, Germany; 3 Inst. Lab Animal Sciences and Welfare, Jena University Hospital—Friedrich Schiller University, Jena, Germany; 4 Institute of Medical Statistics, Computer Sciences and Documentation Science, Jena University Hospital—Friedrich Schiller University, Jena, Germany; 5 Institute for Biochemistry II, Jena University Hospital—Friedrich Schiller University, Jena, Germany; 6 Institute of Anatomy I, Jena University Hospital—Friedrich Schiller University, Jena, Germany; Fraunhofer Research Institution of Marine Biotechnology, GERMANY

## Abstract

While the cerebral autoregulation sufficiently protects subcortical brain regions during hypoxia or asphyxia, the cerebral cortex is not as adequately protected, which suggests that regulation of the cerebral blood flow (CBF) is area-specific. Hypoxia was induced by inhalation of 5% oxygen, for reoxygenation 100% oxygen was used. Cortical and subcortical CBF (by laser Doppler flowmetry), blood gases, mean arterial blood pressure (MABP), heart rate and renal blood flow were constantly monitored. Low dosed urapidil was used for α1A-adrenergic receptor blockade. Western blotting was used to determine adrenergic receptor signalling mediators in brain arterioles. During hypoxia cortical CBF decreased to 72 ± 11% (mean reduction 11 ± 3%, p < 0.001) of baseline, whereas subcortical CBF increased to 168±18% (mean increase 43 ± 5%, p < 0.001). Reoxygenation led to peak CBF of 194 ± 27% in the subcortex, and restored cortical CBF. α1A-Adrenergic blockade led to minor changes in cortical CBF, but massively reduced subcortical CBF during hypoxia and reoxygenation–almost aligning CBF in both brain regions. Correlation analyses revealed that α1A-adrenergic blockade renders all CBF-responses pressure-passive during hypoxia and reoxygenation, and confirmed the necessity of α1A-adrenergic signalling for coupling of CBF-responses to oxygen saturation. Expression levels and activation state of key signalling-mediators of α1-receptors (NOSs, CREB, ERK1/2) did not differ between cortex and subcortex. The dichotomy between subcortical and cortical CBF during hypoxia and reoxygenation critically depends on α1A-adrenergic receptors, but not on differential expression of signalling-mediators: signalling through the α1A-subtype is a prerequisite for cortical/subcortical redistribution of CBF.

## Introduction

Neurological deficits and impairments are in many cases accompanied by a significant decrease in the patients' quality of life and capability to uphold their former lifestyle, both in regard to private and work-related matters. Since the brain is especially vulnerable to ischemia [1], a variety of medical emergencies can lead to global ischemia and result in neurological damages, for example trauma [2–4], cardiac arrest [1], cerebral stroke [5] and (albeit rarer) traumatic asphyxia [6, 7]. Secondary damage of the brain cells can furthermore occur due to reperfusion injury [8]. The long-term consequences of an ischaemic episode suffered by the brain are mostly determined by the extent of damage sustained by the cerebral cortex [9, 10]. This notion was experimentally confirmed in a sheep model of ischemia, where lesions in the cerebral cortex and striatum were observed earlier than lesions in e.g. the thalamic region [11]. While the primary treatment for all of the above mentioned emergencies is either reoxygenation or, respectively, reperfusion, cerebral autoregulatory mechanisms are able to protect the brain from both hypoperfusion and ischemia through changes in the cerebral blood flow (CBF) up to a certain point [12, 13] albeit the degree of brain protection by autoregulation depends on the underlying cause of damage. Unfortunately, the cerebral autoregulation is not yet fully understood at this point, and neither are its contributing agents and mechanisms. The nitric oxide system has been controversially discussed for its role in the cerebral autoregulation. While the exact extent of its contribution still remains unknown, a number of studies have identified at least a supporting role in cerebral autoregulatory mechanisms [14–16].

A further aspect in regard to cerebral autoregulation that has been widely discussed concerns alpha1 (α1)-adrenergic receptors. Although the exact role of α1-receptors has not yet been identified, previous studies have presented conflicting evidence for their influence on the regulation of the CBF. In a model of human exercise the non-selective α1-receptor antagonist prazosin improved blood flow, indicating α1-receptor mediated contraction [17, 18]. In contrast, in the rat carotid artery α1D-receptor signalling is involved in relaxation [19, 20]. In previous studies we used sheep as an animal model, which are a convenient choice because of their similarity to humans in regard to body weight, blood volume and cerebral anatomy (such as gyration and vascular supply) [21, 22]. These previous investigations revealed the redistribution of CBF during severe blood loss and subsequent reperfusion to be critically dependent on the activity of α1A-adrenergic receptors.

We used the same animal model of adult sheep to test whether the redistribution of CBF is dependent on α1A-adrenergic receptors under conditions of ischemia as well. Region-specific changes of CBF during controlled hypoxia and subsequent reoxygenation were measured continuously and the role of α1A-receptors was examined by utilising the selective antagonist urapidil. Furthermore, we analysed the expression of mediators of α1-receptor signalling.

## Materials and methods

### Animal care and surgical instrumentation

All procedures were approved by the Thuringia Animal Welfare Committee (Bad Langensalza; permission number: TVA 02-60/10; valid from 20 December 2010 until 20 December 2015) and conducted in accordance to the ARRIVE guidelines [23]. Thirteen 2–6 year old female Merino long wool sheep, weighing 89.5 ± 8.3 kg, underwent surgery. All animals remained under general anesthesia for the entire duration of the experiment and did not experience any pain or distress. The anesthesia and the surgical approach protocol were described previously [21, 24]. Briefly, anesthesia was started by intramuscular injection of 10–15 mg·kg−1 ketamine (Ketamin-Hydrochlorid®, Pfizer, Berlin, Germany) and 0.2 mg·kg−1 midazolam (Midazolam-Hameln®, Hameln Pharmaceuticals, Hameln, Germany). After orotracheal intubation anesthesia was maintained by inhalation of 1.5% isoflurane (Isofluran–Actavis®, Actavis, Langenfeld, Germany) in 100% oxygen, except during the hypoxic phase of the experiment. We continuously monitored region specific changes of cortical and subcortical CBF by laser Doppler flowmetry [25]. Measurement of renal blood flow (RBF) by a flow probe, mean arterial blood pressure (MABP), electrocardiogram (ECG) and heart rate (HR), and drawing of blood samples were described previously [21, 24]. Briefly, single-fiber laser Doppler flow probes (diameter 400 μm, Moor, Devon, UK) for the continuous monitoring of capillary CBF changes were stereotactically inserted 2 mm into the parietal cortex and 2.7 cm into the subcortex (caudal part of the thalamus, verified post mortem). CBF was recorded at a sample rate of 40/s with a laser Doppler flowmeter (DRT4, Moor, Devon, UK). After the experiment animals were euthanized by intravenous injection of pentobarbital sodium (Narcoren, Merial, Halbergmoos, Germany) as described previously [26, 27].

### Hypoxia and reoxygenation

Hypoxia was induced by inhalation of 5% oxygen and 95% nitrogen and was maintained for 14 minutes. After this period, we intialised the reoxygenation with 100% oxygen over 20 minutes under constant monitoring of breathing and HR, blood pressure, blood gas concentrations, body temperature and oxygen saturation.

### Analysis of blood gases

Blood gas samples were taken before the start of oxygen withdrawal and in two-minute intervals during hypoxia and reoxygenation. Samples were measured on a standard clinical blood gas analyzer (ABL 600, Radiometer GmbH, Willich, Germany).

### α1A-adrenergic blockade

Five randomly assigned sheep underwent the same experimental procedure with α1A-adrenergic receptor blockade with urapidil (Urapidil-Phamore^®^, Phamore, Ibbenbüren, Germany). After 10 min of baseline recordings, an initial bolus of 8 mg urapidil was injected, followed by continuous infusion at a rate of 8 mg/h over the remaining observation period. Previous experiments determined this dosage to be the maximal dose not affecting MABP, HR and RBF [21]. Hypoxia was initiated 20 min after urapidil application, allowing for observation of effects caused by urapidil alone.

### Sample preparation and western blotting

Both cortical and subcortical brain arterioles were harvested from 13 age-matched control sheep after euthanasia that did not undergo the experimental procedure. However, they were anesthetized like the animals undergoing these experiments. These control animals were euthanized with phenobarbital and vessels isolated immediately in order to analyse possible regional differences in mediators controlling vascular smooth muscle tone–undisturbed by the experimental procedure itself: Third branches of region-specific arterioles were snap frozen in liquid nitrogen. Sample preparation and Western blotting protocol were described previously [28]. The antibodies used were initially generated against the corresponding human antigens, are known to react against orthologs in various other mammals and recognize bands of expected molecular weight in sheep samples. Targets were nitric oxide synthases (NOS), cyclic AMP-responsive element-binding protein 1 (CREB) and extracellular signal-regulated kinases (ERK). Activation state was detected by use of phosphorylation site-specific antibodies (indicated by the prefix P-). These include: anti-NOS1 (nNOS; sc-8309 (H-299)) and anti-NOS3 (eNOS; sc-8311 (H-159)), both from Santa Cruz Biotechnology (Dallas, TX, USA); anti-phospho-eNOS (P-Ser1177; 9570), anti-CREB (rabbit monoclonal 48H2; 9197), anti-phospho-CREB (P-Ser133; rabbit monoclonal 87G3; 9198), anti-ERK1/2 (rabbit monoclonal 137F5; 4695) and anti-phospho-ERK1/2 (P-Thr202/Tyr204; rabbit monoclonal D13.14.4; 4370), all from Cell Signaling Technologies (Frankfurt am Main, Germany). These primary antibodies were all used in a final dilution of 1:1000. Mouse anti-β-actin (1:5.000; AC-15, A5441; Sigma-Aldrich, Taufkirchen, Germany) was used for normalization. Secondary antibodies were goat anti-rabbit-IgG-HRP (1:5.000; sc-2004) and goat anti-mouse-IgG-HRP (1:5.000; sc-2031) (both from Santa Cruz Biotechnology). Enhanced chemiluminescence imaging was done on a GBox iChemiXL (Syngene, Cambridge, UK). A reference sample (produced by mixing nine brain vessel extracts) was included in each gel for the comparison of protein expression in different gels. Therefore all quantitative data are normalized to that reference.

### Data analysis

Blood gases and lactate were measured on a standard clinical blood gas analyzer (ABL 600, Radiometer GmbH, Willich, Germany). CBF was recorded using a laser Doppler flowmeter (DRT4, Moor, Devon, UK) as described previously [25]. CBF values are given in arbitary units. All biophysical variables were amplified and sampled at 1000 Hz using a data acquisition and analysis system (Labchart Pro7, ADInstruments, Spechbach, Germany). MABP was calculated and HR was triggered from R waves continuously. Then, all parameters were averaged over five seconds.

### Statistical analysis

Linear mixed regression models were fitted to compare the trend of blood gases, vital parameters, cortical and subcortical CBF between both groups. This approach allows accounting for the correlation of the repeated measurements of an animal. Independent variables of the models are group (urapidil/control) and time of measurement, as well as their interaction. For each condition (hypoxia, no hypoxia) a separate model was calculated. Regression coefficients of the models were presented to describe the average change of the parameters per minute. The significance level was set to α = 0.05. Analyses were performed with SPSS 22.0 (IBM, New York, NY, USA). Data are given in mean differences ± standard error (SEM) or describe a trend over the time of the represented variable each per minute. Linear regression was used to analyse dependences of blood flow and blood pressure, oxygen saturation and MABP and oxygen saturation and HR, respectively. Protein expression data were compared with the Wilcoxon signed rank test for paired samples. These analyses were done with SigmaPlot 13.0 (Systat Software, Erkrath, Germany). *p*-Values of less than 0.05 were considered statistically significant.

## Results

### Effects of hypoxia and reoxygenation on arterial blood gases and vital parameters

**Fig 1:**
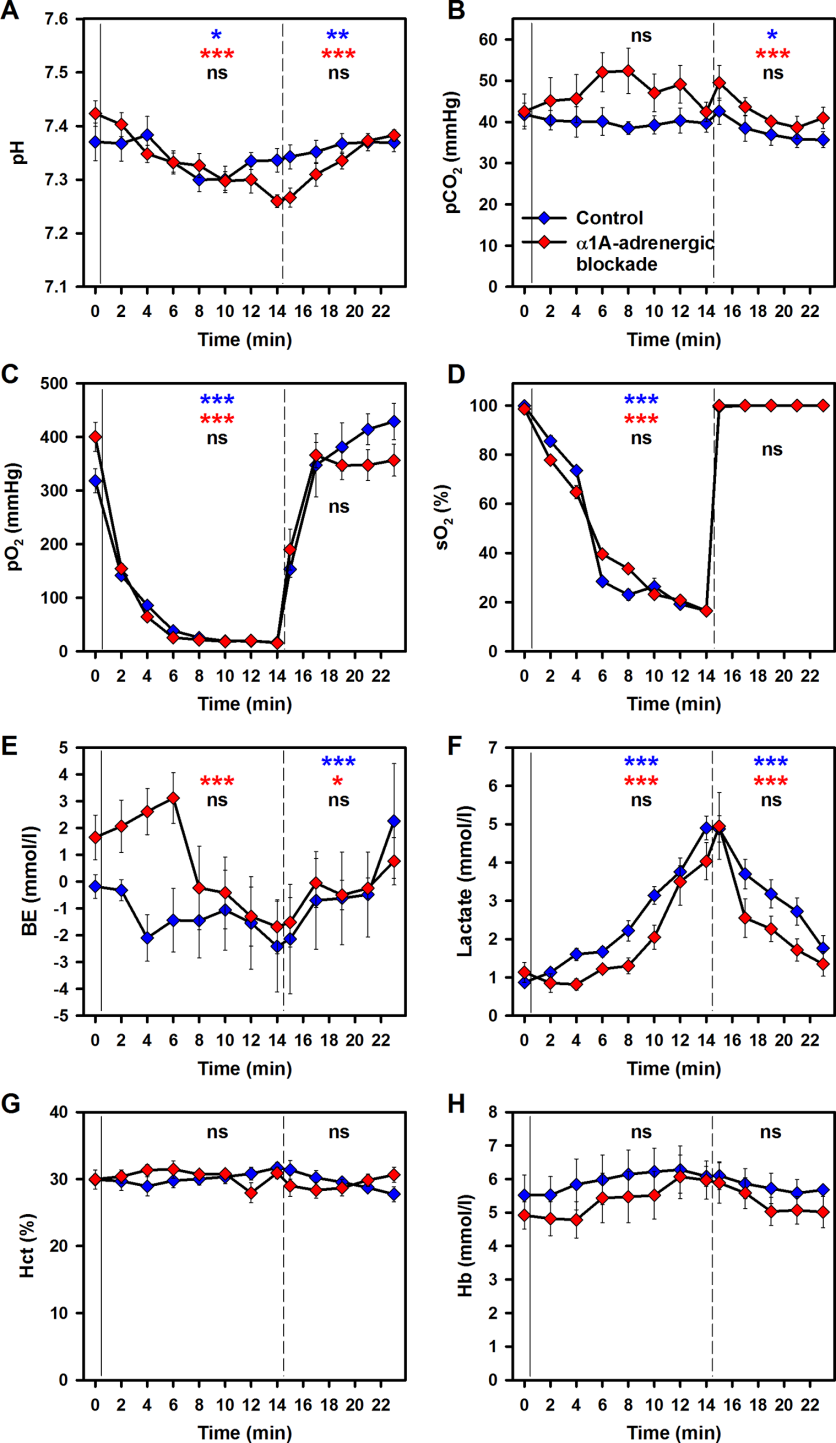
Effects of hypoxia and reoxygenation on arterial blood parameters. Values are given for baseline (min 0), during hypoxia (min 2 to min 14) and during reoxygenation (min 15 to min 23) in controls (blue) and after α1A-adrenergic blockade (red) for (A) pH, (B) partial pressure of carbon dioxide (pCO_2_), (C) partial pressure of oxygen (pO_2_), (D) oxygen saturation (sO_2_, measured with a clinical blood gas analyzer), (E) base excess (BE), (F) lactate, (G) hematocrit (Hct) and (H) hemoglobin (Hb). Through lines separate baseline and hypoxia; dashed lines separate hypoxia and reoxygenation. Means ± SEM; * p < 0.05, ** p < 0.01 and *** p < 0.001 for trend, indicated separately for hypoxia and reoxygenation, respectively; black symbols indicate differences between treatment groups; n.s., not significant.

Arterial blood gases, lactate, hematocrit and hemoglobin were within the physiological range before the onset of controlled hypoxia and after reoxygenation (Fig 1).

During the hypoxic intervention, the pH decreased significantly by 0.004 ± 0.001 per minute in the control group (p = 0.012) and by 0.01 ± 0.001 per minute under alpha1A(α1A)-adrenergic blockade with urapidil (p < 0.001) (Fig 1A). During reoxygenation the pH values increased by 0.003 ± 0.001 per minute in the control group (p = 0.003) and by 0.01 ± 0.001 per minute in the urapidil-treated group (p < 0.001). No differences exist between the groups (Fig 1A).

There was a tendency for increased pCO_2_ values in the urapidil-treated group under hypoxic intervention. However, this was not statistically significant. During reoxygenation the pCO_2_ decreased by 0.9 ± 0.4 mmHg (p = 0.026) per minute in the control group and by 0.7 ± 0.2 mmHg (p = 0.001) per minute in the urapidil-treated group. No significant differences could be observed between the groups under hypoxic intervention and reoxygenation, respectively (Fig 1B).

Hypoxia decreased the pO_2_ by 17.5 ± 2.1 mmHg per minute in the control group (p < 0.001) and by 20.1 ± 2.7 per minute in the urapidil-treated group (p < 0.001). During reoxygenation, pO_2_ recovered to baseline values. No differences exist between the groups (Fig 1C). In parallel, hypoxia decreased the sO_2_ by 6.3 ± 0.5% per minute in the control group (p < 0.001) and by 5.9 ± 0.3% per minute in the urapidil-treated group (p < 0.001). During reoxygenation, the sO_2_ values increase within the first minute to baseline levels in both groups. No differences exist between the groups (Fig 1D).

For BE (Fig 1E), lactate (Fig 1F), Hct (Fig 1G) and Hb (Fig 1H) also no significant differences exist between the groups during hypoxia and reoxygenation. A transient decrease of BE during hypoxia was mirrored by the transient increase of lactate.

**Fig 2:**
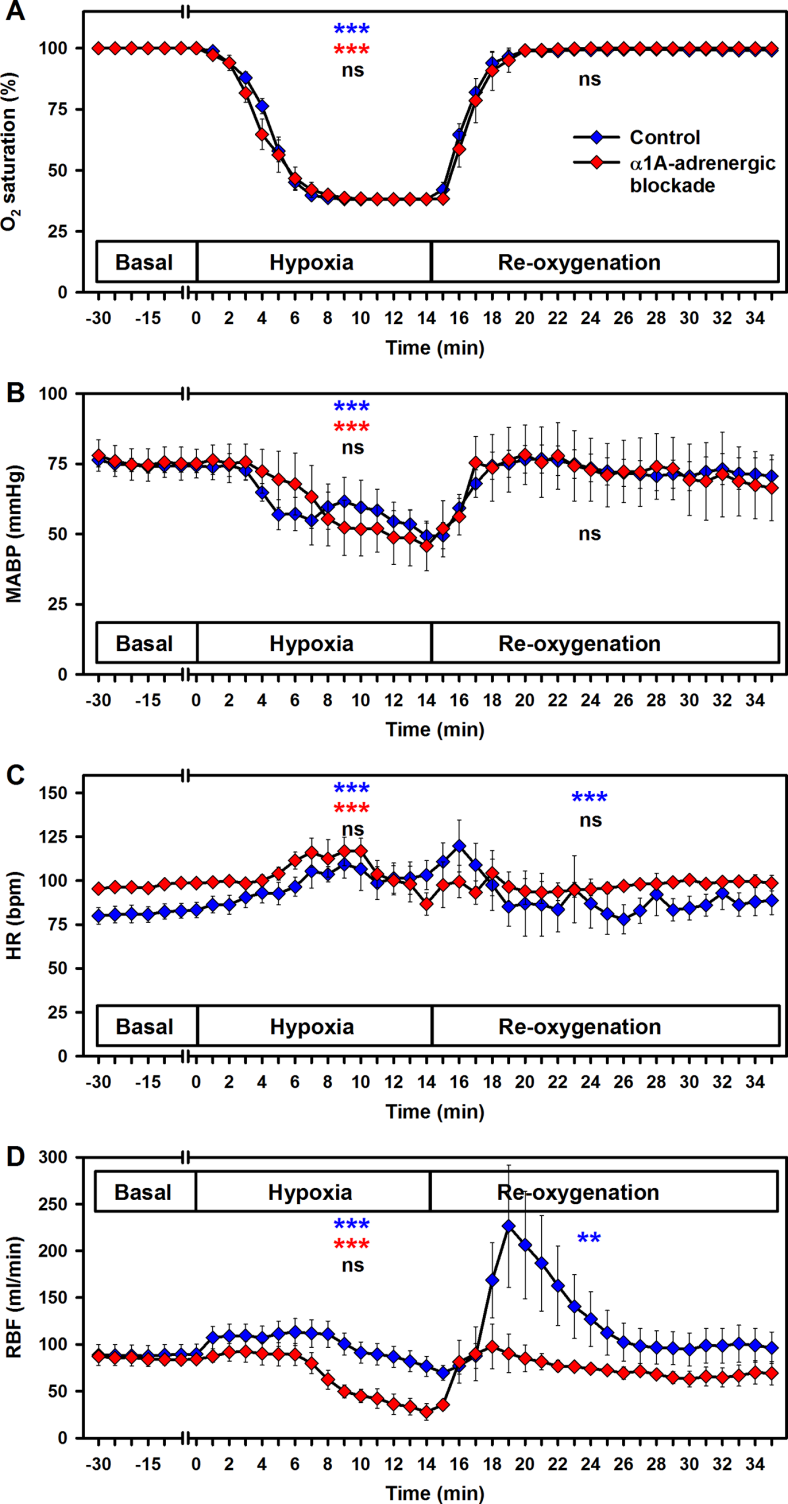
Effects of hypoxia and reoxygenation on vital parameters. (A) oxygen saturation (measured by pulse oximetry), (B) mean arterial blood pressure (MABP), (C) heart rate (HR) and (D) renal blood flow (RBF). Controls in blue and α1A-adrenergic blockade in red. Means ± SEM; * p < 0.05, ** p < 0.01 and *** p < 0.001 for trend, indicated separately for hypoxia and reoxygenation, respectively; black symbols indicate differences between treatment groups; n.s., not significant.

Pulse oximetry was utilised to monitor the hypoxia during the experiments (Fig 2A). It has to be noted that technical limitations prevented accurate measurement of values below 38%. Nevertheless, the data for sO_2_ correlate with those from the analyses of blood gases (Fig 1D).

Hypoxia induced a significant decrease in mean arterial blood-pressure (MABP) from 74 ± 2 mmHg at baseline to 49 ± 5 mmHg by 1.6 ± 0.4 mmHg per minute in the control group (p < 0.001) (Fig 2B). The average of the MABP-reduction was 11 ± 2.3 mmHg compared to the baseline (p < 0.001). Subsequent reoxygenation increased the MABP by 0.3 ± 0.1 mmHg per minute (p = 0.016), to 71 ± 6 mmHg until the end of the observation period.

The heart rate (HR) increased slightly to on average 9 ± 3.1 beats per minute (bpm) higher values compared to the baseline (p = 0.002) at a rate of 1.5 ± 0.4 bpm per minute (p < 0.001) during hypoxia (Fig 2C). Reoxygenation led to a decrease to baseline with 1 ± 0.3 bpm per minute (p < 0.001). Renal blood flow (RBF) increased on average 1.8 ± 0.4 ml/min per minute (p < 0.001) during hypoxia (Fig 2D). Moreover, reoxygenation induced an additional, transient increase with 2.5 ± 0.8 ml/min per minute (p = 0.003) in the control group.

### Effects of hypoxia and reoxygenation on CBF

**Fig 3:**
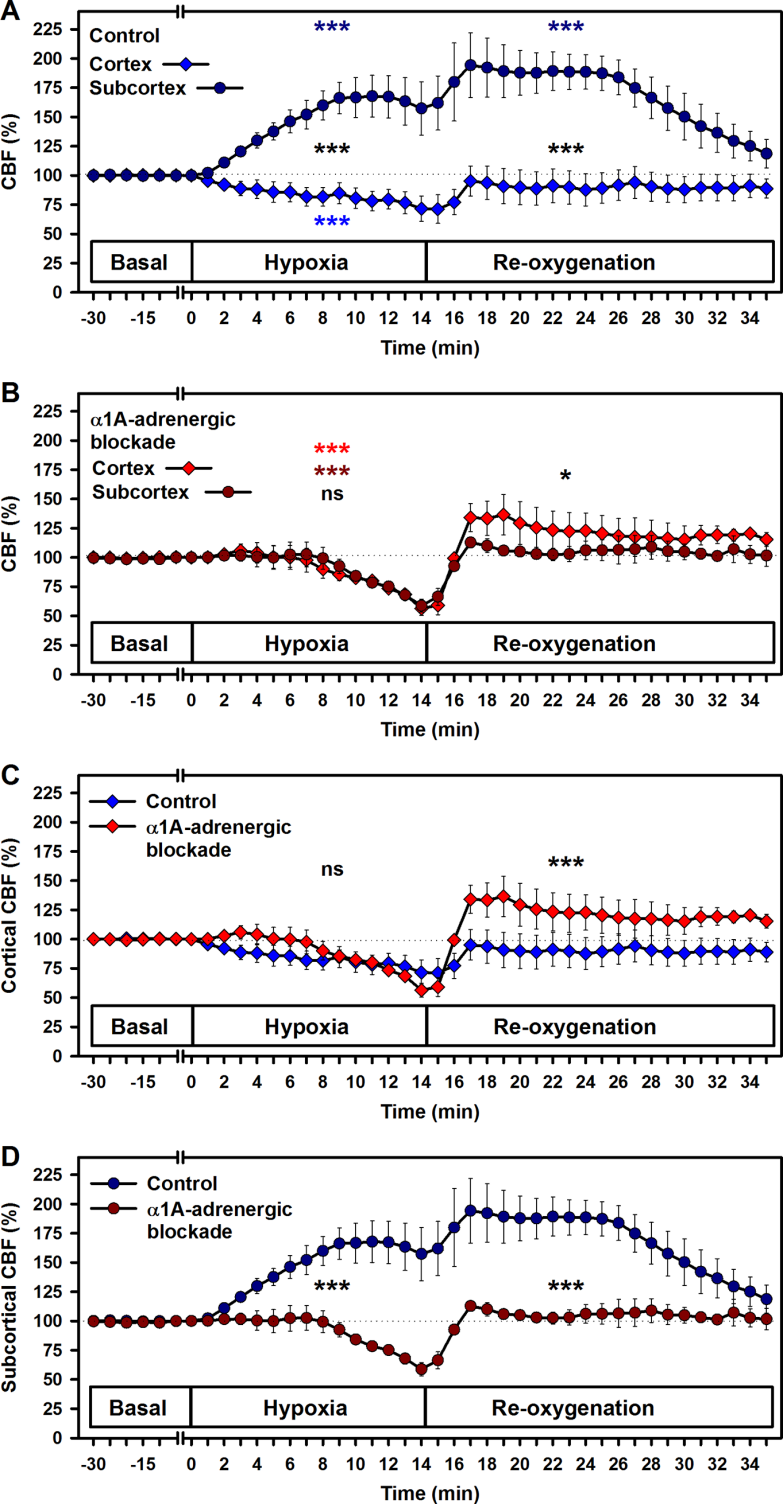
Effects of hypoxia and reoxygenation on cortical and subcortical cerebral blood flow. Comparison of cortical and subcortical cerebral blood flow (CBF) in the control group (A) and under α1A-adrenergic blockade (B). Comparison of controls and α1A-adrenergic blockade for (C) cortex and (D) subcortex. Means ± SEM; * p < 0.05, ** p < 0.01 and *** p < 0.001 for trend, indicated separately for hypoxia and reoxygenation, respectively; black symbols indicate differences between treatment groups; n.s., not significant.

The effects on cortical and subcortical cerebral blood flow (CBF) were significantly different during induced hypoxia and the reoxygenation phase (p < 0.001, Fig 3A). Cortical CBF declined steadily to 72 ± 11% at the end of the hypoxia period (*p* < 0.001), with a mean difference of 11 ± 3% compared to the baseline (p < 0.001). The slope of the cortical CBF decrease was 1.5 ± 0.2% per minute (p < 0.001). During reoxygenation the cortical CBF quickly (within 2 minutes) increased to 95 ± 13% and remained constant until the end of the observation period.

In contrast, subcortical CBF rose steadily from baseline to 168 ± 18% at 10 minutes of hypoxia (p < 0.001). During the hypoxic intervention in the control group, the subcortical CBF increased significantly (mean difference = 43.2 ± 5.4%, p < 0.001) compared to the baseline at a rate of 5.1 ± 0.5% per minute (p < 0.001). During the initial 10 minutes of reoxygenation the subcortical CBF increased even slightly above the values measured under hypoxia, resulting in a mean difference of 65.3 ± 5.3% (p < 0.001) compared to the baseline level. Thereafter, subcortical CBF dropped towards baseline values within another 10 minutes. On average, reoxygenation significantly decreased the subcortical CBF by 3.2 ± 0.5% per minute in the control group (p < 0.001). The differences in cortical and subcortical CBF, respectively, were significant over the complete observation period (p < 0.001) (Fig 3A).

### Effects of α1A-adrenergic blockade

For α1A-adrenergic blockade a low dosage of urapidil was used, which did not affect oxygen saturation, MABP and HR during baseline recordings (minutes -20 to 0 in the figure time-axes), and during hypoxia and subsequent reoxygenation, respectively: sO2 was almost indistinguishable from that of control animals (Fig 2A). Hypoxia induced a significant decrease in MABP of 13 ± 2.4 mmHg (p < 0.001) compared to the baseline and by 2.5 ± 0.3 mmHg per minute (p < 0.001) (Fig 2B). The HR increased 19.9 ± 3.1 bpm (p < 0.001) from baseline (Fig 2C). During hypoxia and reoxygenation, no significant differences of sO_2_, MABP and HR were observed between the groups (Fig 2A–2C).

Analysis of the RBF revealed significant differences during the observation period in the urapidil-treated group as compared to the control group. RBF decreased significantly from 84 ± 4 ml/min at baseline to 28 ± 9 ml/min during hypoxia by 5.1 ± 0.4 ml/min per minute (p < 0.001), whereas reoxygenation restored RBF to baseline values without hyperperfusion as seen in control animals (Fig 2D). During hypoxia, average RBF was significantly lower in the urapidil-treated group than in the control group (mean difference 29.6 ± 12.1 ml/min, p = 0.015) compared to the baseline.

In animals treated with urapidil the decrease of cortical CBF to 56 ± 6% (p < 0.001) as compared to the baseline level by 3 ± 0.3% per minute (p < 0.001) followed a similar pattern as observed in the decrease of subcortical CBF to 55 ± 7% (p < 0.001) by 2.8 ± 0.4% per minute during hypoxia (Fig 3B). Subsequent reoxygenation restored subcortical CBF to baseline, which was not exceeded significantly over time. α1A-adrenergic blockade exerted its effect on the cortical CBF mainly during reoxygenation in form of a moderate hyperperfusion (Fig 3B and 3C). During hypoxia, no significant differences of cortical CBF exist between both groups.

Administration of urapidil triggered a significantly stronger reaction in the subcortex than in the cortex, both during hypoxia and reoxygenation, which is evidenced by the drastic decrease of the hyperperfusion that could otherwise be observed under control conditions (Fig 3D). During the hypoxic intervention, the subcortical CBF was significantly lower in the urapidil-treated group than in the control group (mean difference 51.3 ± 8.7%, p < 0.001). On average, hypoxia significantly decreased the subcortical CBF by 2.8 ± 0.4% per minute in the urapidil-treated group (p < 0.001). The reduction of subcortical CBF in the urapidil-group continued through reoxygenation (mean difference 62.2 ± 8.7%, p < 0.001) (Fig 3D).

**Fig 4:**
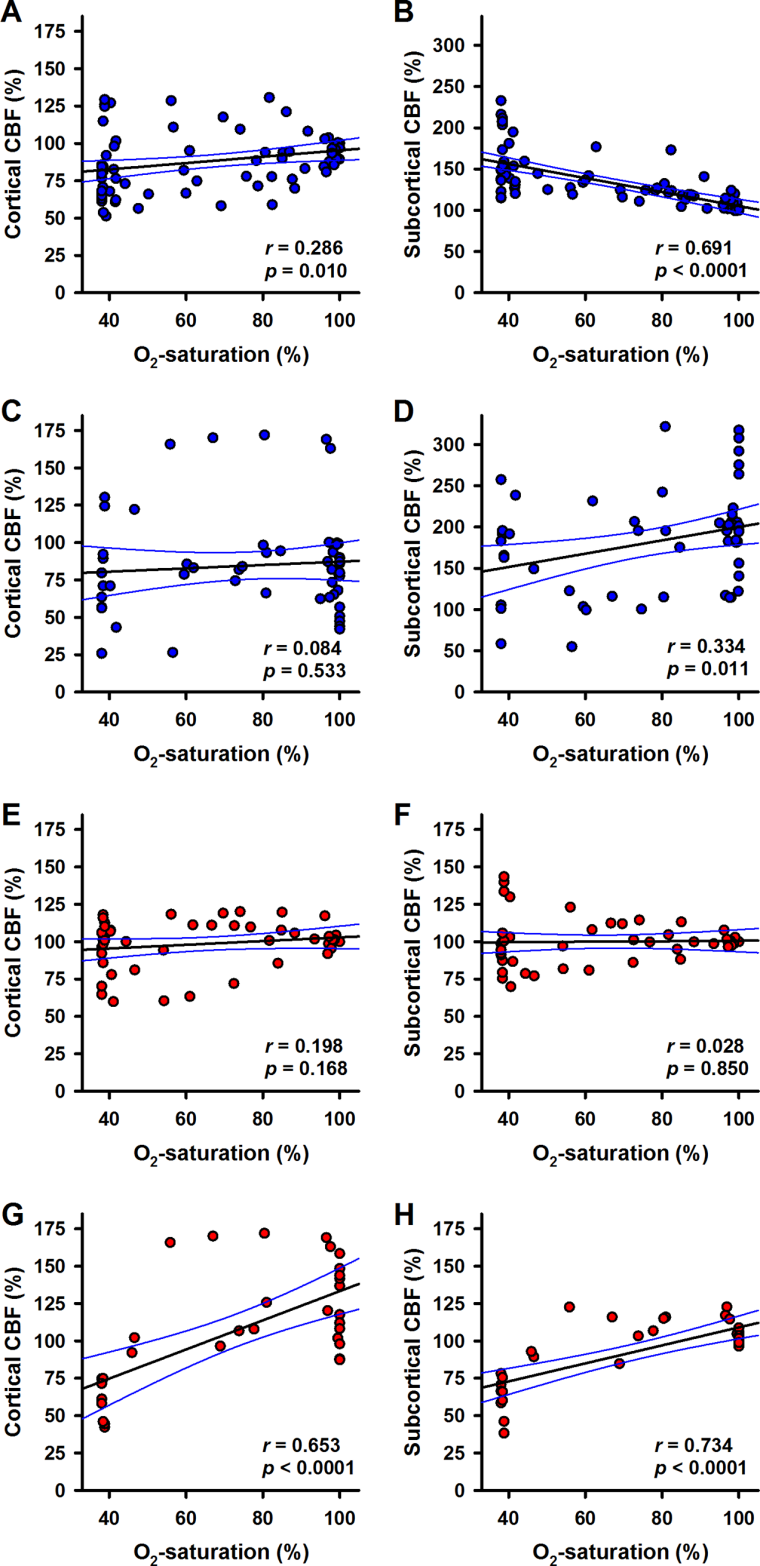
Correlation of blood flow and oxygen saturation. Cortical or subcortical CBF in the control group (blue symbols) were plotted against oxygen saturation (sO_2_) during hypoxia (A, B) and for the reoxygenation phase (C, D), respectively. The effect of α1A-adrenergic blockade (red symbols) on the relationships of blood flow and sO_2_ during hypoxia (E, F) or during reoxygenation (G, H) is plotted analogously. Linear regression was calculated for each data set. Best fit lines (black lines) and 95% confidence intervals (blue lines) are plotted. Correlation coefficients (*r*) and *p*-values are given in the respective panels.

Under α1A-adrenergic blockade correlation analysis for cortical CBF and subcortical CBF, respectively, in relation to oxygen saturation revealed no differences between both brain regions. During hypoxia, there was no correlation in both regions (Fig 4E and 4F), whereas oxygen saturation was correlated with CBF during reoxygenation (Fig 4G and 4H; p < 0.0001 for both data sets).

**Fig 5:**
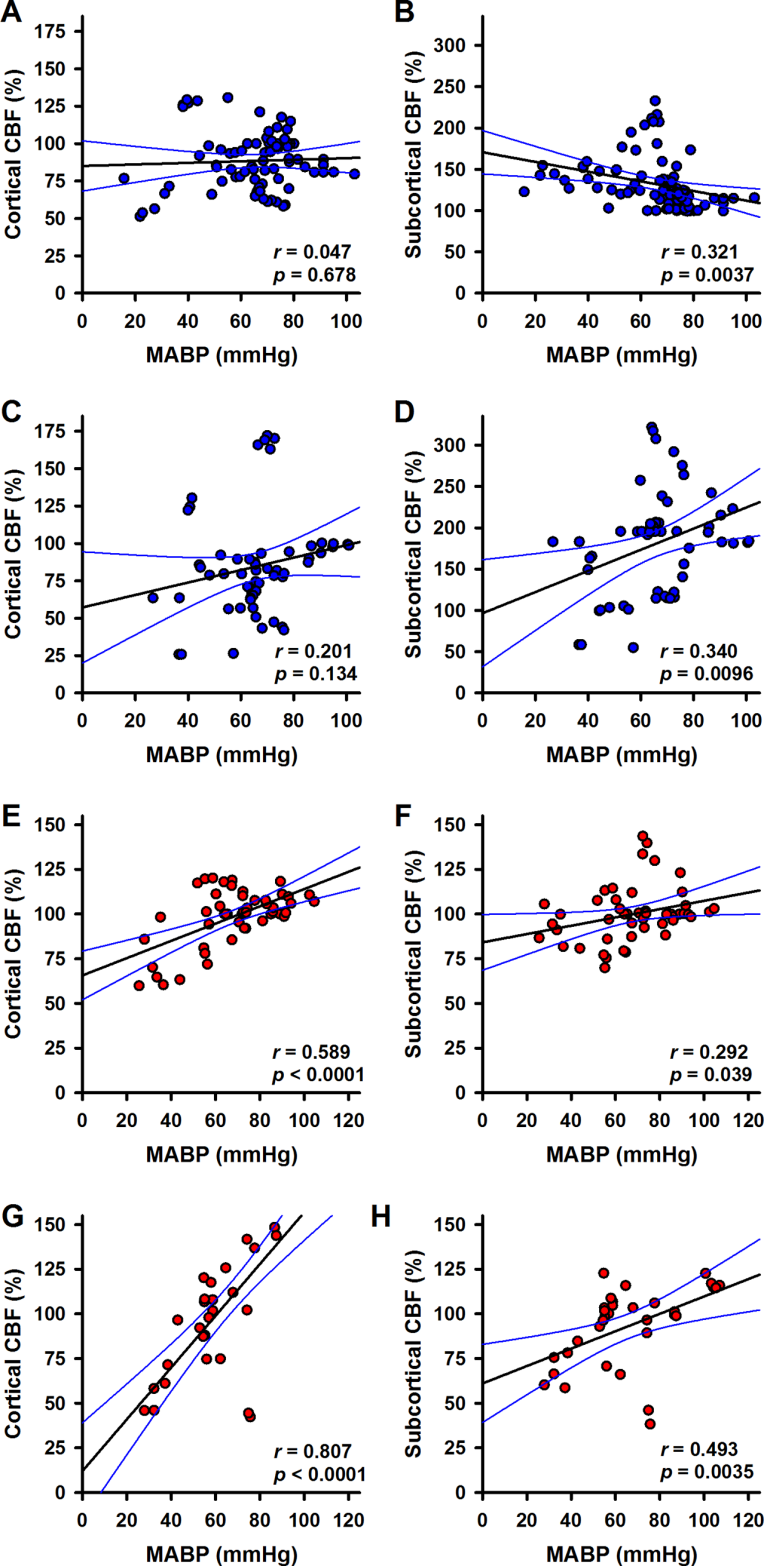
Correlation of blood flow and MABP. Cortical or subcortical CBF in the control group (blue symbols) were plotted against MABP during hypoxia (A, B) and for the reoxygenation phase (C, D), respectively. The effect of α1A-adrenergic blockade (red symbols) on the relationships of blood flow and MABP during hypoxia (E, F) or during reoxygenation (G, H) is plotted analogously. Linear regression was calculated for each data set. Best fit lines (black lines) and 95% confidence intervals (blue lines) are plotted. Correlation coefficients (*r*) and *p*-values are given in the respective panels.

**Fig 6:**
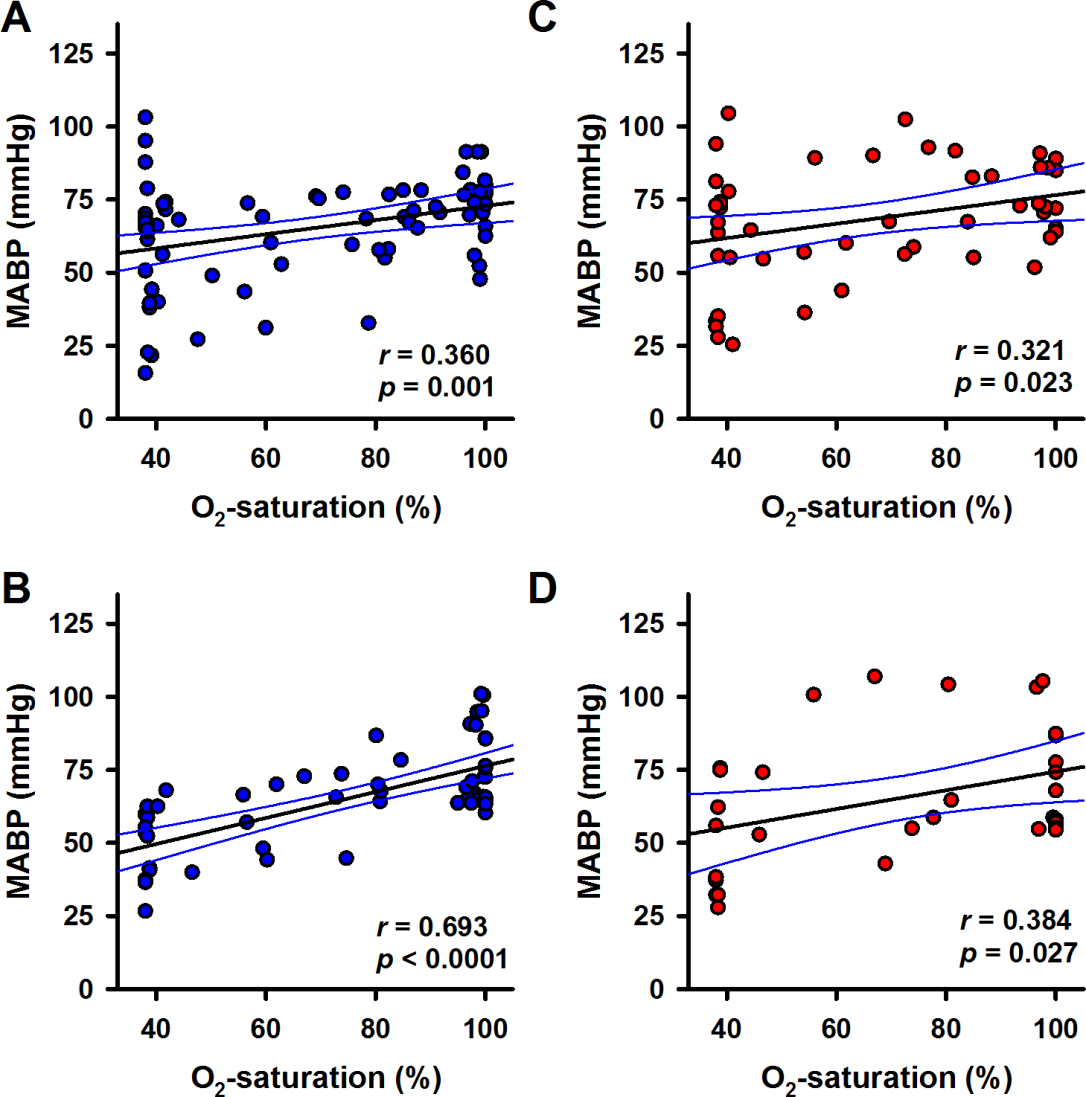
Correlation of and MABP and sO_2_. MABP in the control group (blue symbols) was plotted against sO_2_ during hypoxia (A) and for the reoxygenation phase (B), respectively. The effect of α1A-adrenergic blockade (red symbols) on the relationships of MABP and sO_2_ during hypoxia (C) or during reoxygenation (D) is plotted analogously. Linear regression was calculated for each data set. Best fit lines (black lines) and 95% confidence intervals (blue lines) are plotted. Correlation coefficients (*r*) and *p*-values are given in the respective panels.

Interestingly, there was a significant correlation of cortical and subcortical CBF, respectively, and MABP during hypoxia (Fig 5E and 5F; p < 0.0001 and p = 0.039, respectively), as well as during reoxygenation (Fig 5G and 5H; p < 0.0001 and p = 0.0035, respectively). Under α1A-adrenergic blockade, a correlation between MABP and oxygen saturation was found during hypoxia (p = 0.023, Fig 6C) as well as reoxygenation (p < 0.027, Fig 6D).

### Correlations of blood flow, oxygen saturation and MABP

In order to examine a potential relationship between blood flow and oxygen saturation, region-specific CBF was plotted versus oxygen saturation. Data were separately plotted for the hypoxic phase (Fig 4A and 4B) and for the reoxygenation phase (Fig 4C and 4D). Linear regression analysis showed significant correlation of cortical (p = 0.01) and subcortical (p < 0.0001) CBF and oxygen saturation during hypoxia. During reoxygenation only subcortical CBF correlated with oxygen saturation (p = 0.011).

As MABP decreased during hypoxia, also a possible relationship of blood flow and MABP was tested by plotting region-specific CBF versus MABP. Data were plotted for the hypoxic phase (Fig 5A and 5B) and for the reoxygenation phase (Fig 5C and 5D) separately. There was significant correlation of subcortical CBF and MABP during hypoxia (p = 0.0037) or reoxygenation (p = 0.0096). In contrast, there was no correlation of cortical CBF and MABP (neither during hypoxia nor during reoxygenation). In addition, the correlation of MABP and oxygen saturation was confirmed for hypoxia (p = 0.001, Fig 6A) and reoxygenation (p < 0.0001, Fig 6B).

### Expression and activation state of signalling mediators of smooth muscle tone

**Fig 7:**
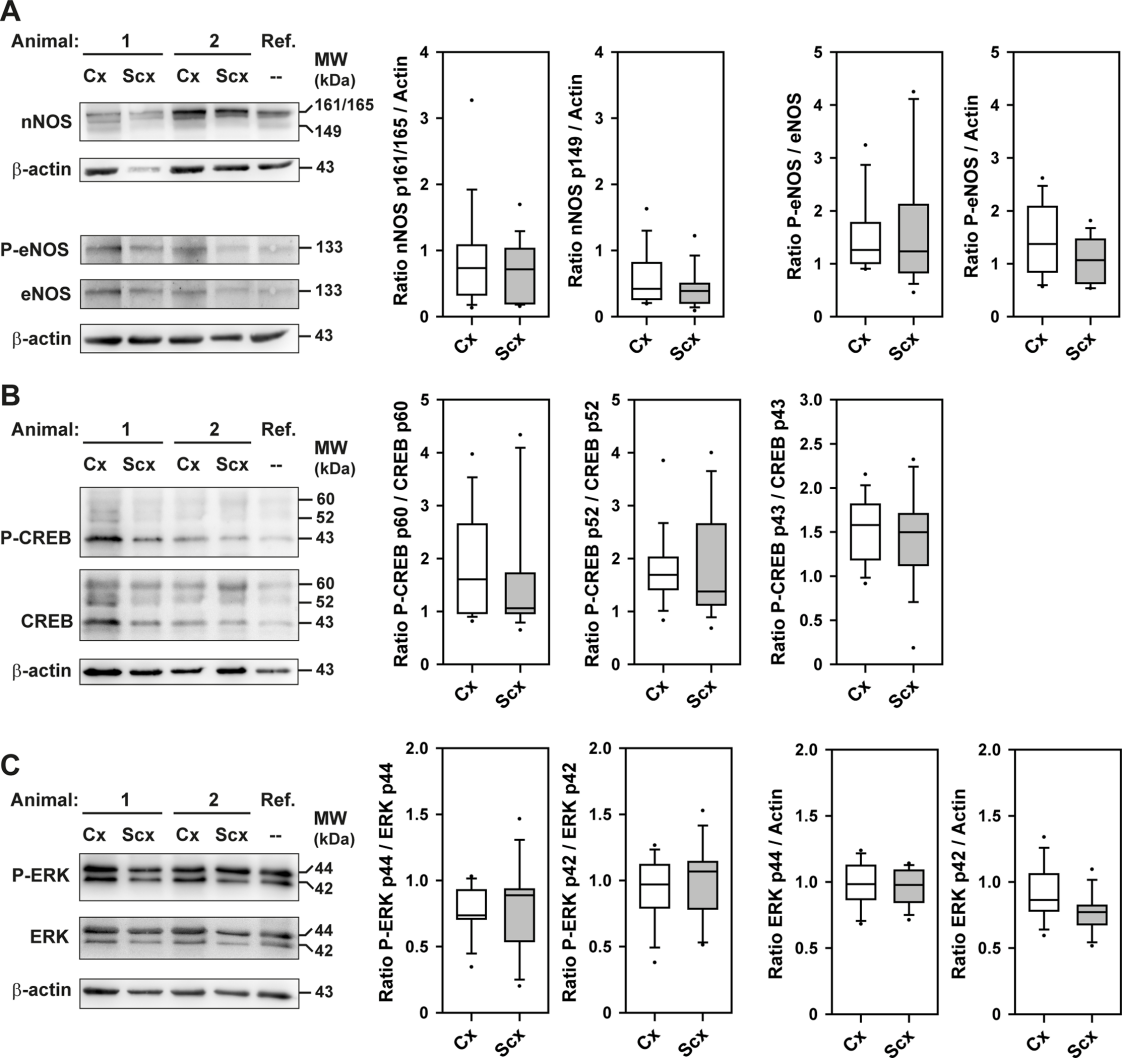
Expression and activation state of signalling mediators involved in regulation of smooth muscle tone. Western blot analysis was performed for cortical and subcortical brain arterioles from 13 sheep, as described in the methods section. Differences of expression levels of signalling proteins involved in control of smooth muscle tone: (A) nNOS, P-eNOS and eNOS-protein, (B) P-CREB and CREB protein, (C) P-ERK and ERK protein was detected in relation to β-actin. As these data were not normally distributed they are presented as box plots, where boxes represent 25th and 75th percentiles, respectively. Medians are indicated by horizontal lines. Whiskers indicate 10th and 90th percentiles, respectively. 1+2, samples from two different sheep; Cx, cortex; Scx, subcortex; AU, arbitrary units; Ref. reference sample.

Possible differences of expression levels or the activation state of signalling proteins involved in the control of smooth muscle tone were analysed by Western blotting. Samples were prepared from brain arterioles from both cortex and subcortex of 13 age-matched control sheep. The neuronal nitric oxide synthases (nNOS)-antibody detected three bands of 161/165 kDa and 149 kDa (identified by molecular weight of human orthologs), whereas the endothelial nitric oxide synthases (eNOS)-antibodies recognized one band of the expected molecular weight of 133 kDa (Fig 7A). The cyclic AMP-responsive element-binding protein 1 (CREB)-antibodies reacted with a band of expected molecular weight of 43 kDa and two putative splice variants of 52 kDa and 60 kDa (Fig 7B). The extracellular signal-regulated kinases (ERK)-antibodies detected the highly conserved paralogs ERK1 and ERK2 (44 kDa and 42 kDa, respectively; Fig 7C). Differences of expression levels in relation to β-actin could not be found between cortical and subcortical arterioles. In addition, no region-specific difference of phosphorylation of eNOS, CREB and ERK1/2 was detectable.

## Discussion

From the results of this study we draw the conclusion that, during states of hypoxia, subcortical brain regions exhibit a superior protection through the cerebral autoregulation as compared to the cerebral cortex, which can be attributed to region-specific regulation of the CBF.

### Cerebral blood supply in humans and sheep

A reason for the use of a sheep model for translational CBF research is their remarkable anatomical similarity to the human brain [29, 30]. Taking the importance of anatomical comparability, the sheep’s large size and gyrencephalic pattern, dense white matter tracts, strong fibrous dura mater and tentorium cerebelli [31] recommend it for the use in CBF research. Its neurovascular anatomy is very similar to that of humans [32], with the exception of an extradural rete mirabile in young sheep [33].

A more detailed comparison of the vascularization of humans and sheep, respectively, is provided in Table 1. The most significant contributors to the perfusion of the cerebral cortex are the middle cerebral artery and the anterior cerebral artery, which are supplied by the internal carotid artery in both humans [34] and sheep [35]. Therefore, flow probes for measurement of cortical CBF were placed in (and arterioles for western blotting were taken from) this supply region, i.e. the frontoparietal cortex.

**Table 1:** Cerebral blood supply in humans and sheep.

Humans	Sheep
**Cortical cerebral perfusion**	
Supplied by the internal carotid artery [34]	Supplied by the internal carotid artery [35]
Middle cerebral artery segments rise to perforating lenticulostriate arteries from pre- and post-bifurcation segments, also from the anterior cerebral artery or internal carotid artery [36]	Rhinencephalon correlates to the anterior perforated substance served by the anterior and middle cerebral artery [35]
**Subcortical / thalamic cerebral perfusion**	
Thalamus supplied by branches of the circle of Willis; a rostral inflow of the internal carotid artery and a caudal inflow of the vertebral artery are interconnected through the posterior communicating artery [37, 39]	Thalamic blood supply is very similar to humans [32]
Thalamus located at the boundary of both arterial systems [41]. 59% of blood supplied from the posterior cerebral artery and 35% from the posterior communicating artery [47]	Obliteration of the internal carotid artery after 12–18 months [44]
Posterior arterial supply of the thalamus by the posterior cerebral artery through posterior choroidal arteries [37]	
Choroidal arteries primarily function as suppliers of the choroid plexus [38]	
Blood supply of anterior, medial and posterior thalamus through a single artery from the posterior communicating artery possible (anterior thalamo-perforating artery) [40, 41, 42]	
Blood supply of the lateral thalamus by the medial cerebral artery possible via lenticulostriate arteries [43]	
Developmental relevance of a rete mirabile, but does not contribute to the CBF in later life [46]	Extradural rete mirabile [33]. Blood supply through the rete mirabile already concludes after 4 months [44]. Epidural rostral and caudal rete mirabile supplied through branches of the arteria maxillaris, minor contribution to the CBF [45]

In summary (see Table 1 for details), the thalamus is predominately supplied by the posterior circulation system. Therefore, we took care to place the flow probes for measurement of subcortical CBF in the caudal region of the thalamus. Arterioles for western blotting were also taken from this region.

### Clinical implications

During controlled hypoxia, cortical CBF decreased over the course of reduced oxygen supply, whereas subcortical CBF increased in response to the reduction of the oxygen saturation. Obviously, this decrease in the cortical blood flow is the cause for the brain damage that can be observed in patients that have experienced prolonged episodes of hypoxia, a pathologic state that negatively affects the long-term outcome [9, 10].

Although centralisation, as an autoregulatory response, has previously been considered to be able to protect the entirety of the adult brain [36, 37], the fact that CBF responses vary in cortical and subcortical regions, respectively, refutes this previous assumption. In a study with newborn term and preterm sheep, Stonestreet and co-workers found differentially increased CBF in various regions of the brain as a consequence of asphyxia [38]. The difference to our adult model (where cortical CBF is reduced during hypoxia) strongly suggests that the protection from hypoxic damage in the cortex diminishes during aging.

Obviously, studies with a design similar to ours, i.e. massive hypoxia or asphyxia, cannot be done in human subjects for ethical reasons. Nonetheless, even less invasive experimental models have supplied supporting evidence for region-specific CBF responses. Ogoh et al., for example, reported significant increases of vertebral arterial blood flow as compared to unchanged internal carotid arterial blood flow during hypoxia, in conjunction with differences in dynamic cerebral autoregulatory responses, measured through ultrasound technique in conscious human participants [39]. Albeit obvious differences in the severity of the employed experimental hypoxia (90% oxygen saturation in Ogoh et al., 20% in our own model), the results of both experiments nonetheless clearly support the case for a non-uniform distribution of cerebral autoregulatory responses.

This is reminiscent of the situation in hypovolemia, where older studies in different species have shown an increased, unchanged or better preserved CBF in deep brain regions while the cortical CBF was maintained or decreased (overview in [40]). Some studies found no regional CBF differences and one study a smaller decrease of cortical CBF compared to the medulla (overview in [40]). Almost all of these studies used the autoradiography or the microsphere techniques for CBF measurement. Moreover, these studies used small animal models (rats, rabbits, cats, etc.), which may not completely reflect the human situation. However, newer studies with humans [41] or the sheep model [21] indicate that there is a clear distinction between cortical CBF and subcortical CBF under conditions resulting in a reduction of mean arterial blood pressure (MABP). Here, in summary, subcortical CBF, which is mainly supplied by the vertebral arteries, is largely maintained at the cost of cortical CBF, which is mainly supplied by the internal carotid arteries.

This inadequate protection of the cortical brain structures is consequently the cause of the sustained ischemic brain damage during hemorrhage [3, 42] as well as during hypoxia [1, 5–7, 9, 10, 43]. In order to improve the neurological outcomes of hypoxic patients, maintenance of the CBF is therefore vital, as evidenced by the results of our study [2, 3, 42]. Since administration of 100% oxygen was able to ensure a swift restoration of the oxygen saturation, our results accentuate the importance of the therapeutic administration of oxygen as the most basic neuroprotective agent.

The increase in lactate parallel to the decrease of the oxygen saturation reflects a shift to anaerobic metabolism. We measured these parameters in arterial blood and did not evaluate them in venous blood from different brain areas in our experimental design. Nevertheless, the differential CBF-responses to hypoxia indicate preferential damage of the cortex under these conditions, which suffers from reduced perfusion with blood that is already not well oxygenated. In contrast, this reduced oxygen saturation could be compensated at least partially by increased blood flow to the subcortex.

The degree of dependency between subcortical and cortical CBF, respectively, and the oxygen saturation differed significantly. This indicates the involvement of an oxygen-sensitive mechanism controlling the redistribution of the CBF. Indeed, there was a strong inverse correlation between the subcortical CBF and the oxygen saturation, whereas the cortical CBF was positively correlated with the oxygen saturation. The increase in subcortical CBF as a response to a potential damaging stimulus is somewhat unexpected, but correlates with the findings of hypoxia-triggered elevation of blood flow in the vertebral artery, as described by Ogoh et al. [39].

Depending on the experimentel model (rabbit, macaque or piglet) and methods used, previous studies described changes in CBF caused by isoflurane anesthesia in various brain regions [44–46]. In summary, it was concluded that isoflurane reduces cerebrovascular resistance leading to increased cerebral blood volume and resulting in improved brain tissue oxygenation. The data provided in the present study show that despite these effects of isoflurane, there is a dichotomous response of CBF in cortex and subcortex, respectively, in a situation of acute hypoxia. Importantly, we observed a massive increase of CBF under hypoxia in the subcortex, which already at baseline should benefit from a slight increase of CBF triggered by isoflurane [46]. Moreover, there is good agreement with the findings of Terlecki et al. [11] concerning brain damage: they consistently found a higher degree of damage in the cerebral cortex than in the thalamus over a span of 10 to 30 min of ischemia.

The increase of subcortical CBF in response to hypoxia indicates the involvement of a regulatory mechanism, which actively redistributes the blood flow in different brain regions. In a previous study involving severe hypovolemia we were able to provide evidence that the reduction in CBF is significantly less pronounced in the subcortex as compared to the cortex [21]. This apparent discrepancy in the absolute CBF-responses, however, could have potentially been influenced by the total amount of blood available, which was reduced by 50% in our hypovolemia model. This present study, with a constant available blood volume, therefore provides even stronger evidence that there is active redistribution of the available blood volume towards the subcortex, i.e. phylogenetically older brain regions, which control the essential functions of life [47, 48].

### Potential roles of α1-adrenergic receptors

In previous work we found that a low dose of the α1A-adrenergic receptor (α1A-AR) blocker urapidil annihilated the redistribution of CBF during hypovolemia, whereas it did not decrease MABP, RBF and CBF at baseline [21]. This effect was also found during hypoxia. Importantly, α1A-adrenergic blockade renders the CBF-responses of both cortex and subcortex independent from MABP during hypoxia as well as during reoxygenation. Without urapidil the CBF-response of the cortex was independent from the MABP, whereas the subcortical CBF was inversely correlated with MABP during hypoxia. This indicates the involvement of an α1A-AR with high affinity for urapidil in the mechanism triggering redistribution of CBF. These conclusions were mainly drawn from the correlation analyses presented in Figs 4–6. It should be noted that these were based on the same sets of primary data analysed with linear mixed regression models before and therefore provide no independent confirmation of these findings. Another limitation for the interpretation of our findings might be seen in the fact that we could not run a separate control group (treated with urapidil, but without hypoxia). However, this control was built into the experimental setting: Urapidil administration started 20 min before hypoxia was induced. Therefore, we have an inherent control in the same animals.

The correlations of CBF with oxygen saturation of the blood during hypoxia (positive in cortex, negative in subcortex) were lost in animals with α1A-adrenergic blockade. This indicates that α1A-AR-mediated signalling is necessary for coupling of the CBF-responses to the oxygen concentration. Taken together, these results strongly support a central role for α1A-ARs in the mechanism underlying redistribution of CBF.

It is known that hypercarbia leads to increased blood flow (preferentially in subcortical regions of the brain [49]. In fact, mild hypercarbia (albeit not statistically significant) was found in urapidil treated animals only. However, in these animals blood flow in the subcortex is reduced to levels found in the cortex during hypoxia. Therefore, blockade of α1A-adrenergic signaling even antagonizes the CBF-protective effect of hypercarbia.

In our previous work we showed differential expression of α1-ARs in brain samples from cortex and subcortex of sheep, respectively. Expression of α1A-ARs and α1D-ARs was found [21]: α1A-AR-expression was significantly stronger in the cortex than in the subcortex, respectively, whereas the expression levels of the α1D-AR were higher in the subcortex than in the cortex. In the present study as well as in the case of the observed CBF responses to hypovolemia [21], the different expression levels of the α1A-ARs do not perfectly fit to the effect ranges of α1A-adrenergic blockade in the two brain regions. This suggests that additional signalling mediators are involved. This might be at the level of α1D-AR in a way that α1A-ARs are a prerequisite for CBF-redistribution, whereas the amount of α1D-ARs is responsible for the relative increase of CBF in the subcortex.

To get some insight into the potential involvement of important signalling mediators of α1-ARs, expression or activation state, respectively, of nitric oxide synthases (NOS), cyclic AMP-responsive element-binding protein 1 (CREB) and extracellular signal-regulated kinases (ERK) were analysed [15, 50–56]. We found no differences in the expression levels of neuronal NOS, endothelial NOS (eNOS), CREB and ERK1 or ERK2. Inducible NOS was not expressed in the arterioles. No differences in the activation states were detected by use of phosphorylation site-specific antibodies against phospho-Ser1177-eNOS, phospho-Ser133-CREB; and phospho-Thr202/Tyr204-ERK1/2. Taken together, differences in the expression or basal activities of these key mediators of α1-AR signalling are apparently not responsible for the different CBF-responses of cortex and subcortex to hypoxia, respectively.

## Conclusions

In conclusion, there is a pronounced dichotomy between subcortical and cortical cerebral perfusion during hypoxia and reoxygenation. The subcortical over-perfusion during hypoxia is strongly dependent on α1A-AR-signalling, despite its lower expression in arterioles of the subcortex. In the absence of α1A-AR-signalling, both cortical and subcortical CBF become pressure-passive. Our results suggest that blood circulation during severe hypoxia is maintained sufficiently only in phylogenetic older brain subcortical regions, i.e. thalamus, that are necessary for survival.
